# Fine Mapping and Candidate Gene Analysis of Two Major Quantitative Trait Loci, *qFW2.1* and *qFW3.1*, Controlling Fruit Weight in Pepper (*Capsicum annuum*)

**DOI:** 10.3390/genes15081097

**Published:** 2024-08-20

**Authors:** Congcong Guan, Yuan Jin, Zhenghai Zhang, Yacong Cao, Huamao Wu, Daiyuan Zhou, Wenqi Shao, Chuangchuang Yang, Guoliang Ban, Lingling Ma, Xin Wen, Lei Chen, Shanhan Cheng, Qin Deng, Hailong Yu, Lihao Wang

**Affiliations:** 1 Key Laboratory for Quality Regulation of Tropical Horticultural Crops of Hainan Province, School of Tropical Agriculture and Forestry, Hainan University, Haikou 570228, China; 15835295324@163.com (C.G.); 990865@hainanu.edu.cn (S.C.); 2 State Key Laboratory of Vegetable Biobreeding, Institute of Vegetables and Flowers, Chinese Academy of Agricultural Sciences, Beijing 100081, China; 18538580785@163.com (Y.J.); zhangzhenghai@caas.cn (Z.Z.); caoyacong@caas.cn (Y.C.); wuhuamao@caas.cn (H.W.); sdfxzdy@126.com (D.Z.); 15170195399@163.com (W.S.); yangcc503@163.com (C.Y.); bangl245@163.com (G.B.); mlingling0224@163.com (L.M.); wenxin15832338618@outlook.com (X.W.); wanglihao@caas.cn (L.W.); 3 Institute of Vegetables and Flowers, Chongqing Academy of Agricultural Sciences, Chongqing 408113, China; leichennjau@163.com

**Keywords:** pepper (*Capsicum* spp.), fruit weight, QTL mapping, expression analysis, candidate genes

## Abstract

Fruit weight is an important agronomic trait in pepper production and is closely related to yield. At present, many quantitative trait loci (QTL) related to fruit weight have been found in pepper; however, the genes affecting fruit weight remain unknown. We analyzed the fruit weight-related quantitative traits in an intraspecific *Capsicum annuum* cross between the cultivated species blocky-type pepper, cv. Qiemen, and the bird pepper accession, “129−1” (*Capsicum annuum* var. *glatriusculum*), which was the wild progenitor of *C. annuum*. Using the QTL-seq combined with the linkage-based QTL mapping approach, QTL detection was performed; and two major effects of QTL related to fruit weight, *qFW2.1* and *qFW3.1*, were identified on chromosomes 2 and 3. The *qFW2.1* maximum explained 12.28% of the phenotypic variance observed in two F_2_ generations, with the maximum LOD value of 11.02, respectively; meanwhile, the *qFW3.1* maximum explained 15.50% of the observed phenotypic variance in the two F_2_ generations, with the maximum LOD value of 11.36, respectively. *qFW2.1* was narrowed down to the 1.22 Mb region using homozygous recombinant screening from BC_2_S_2_ and BC_2_S_3_ populations, while *qFW3.1* was narrowed down to the 4.61Mb region. According to the transcriptome results, a total of 47 and 86 differentially expressed genes (DEGs) in the candidate regions of *qFW2.1* and *qFW3.1* were identified. Further, 19 genes were selected for a qRT-PCR analysis based on sequence difference combined with the gene annotation. Finally, *Capana02g002938* and *Capana02g003021* are the most likely candidate genes for *qFW2.1*, and *Capana03g000903* may be a candidate gene for *qFW3.1*. Taken together, our results identified and fine-mapped two major QTL for fruit weight in pepper that will facilitate marker-assistant breeding for the manipulation of yield in pepper.

## 1. Introduction

Pepper (*Capsicum* spp., Solanaceae family) is one of the most consumed vegetables worldwide, with a total production of 41.1 million tons that was worth more than USD 375.7 billion in 2021 (FAOSTAT; http://faostat.fao.org/, accessed on 3 March 2024). *Capsicum* originated from Central and South America and has been consumed since at least 6000 BC; it comprises the well-known sweet and hot chili peppers (domesticated) and several wild species [1]. Peppers are used mainly as fresh vegetables and spices, which are cultivated and consumed worldwide. Because of its rich nutrition and its spicy taste, which is different from other vegetables, it is deeply loved by people [2]. It is also used for extracting capsaicin, essential oils, and carotenoids for a variety of medicine, natural colorants, and cosmetics [3].

Increasing production is an essential component of the endeavor to ensure food security, and it is the main goal of almost all vegetable breeding [4]. Yield in the Solanaceae family is a complex trait and is affected by many factors, including the amount of fruit and the dimensions, maturity, and weight of the fruit [5]. However, compared to grain crop species [6], less is known about the genes that underlie the yield in the Solanaceae family [7]. Fruit weight is an important characteristic of Solanaceae, which directly affects the yield of plants and is usually quantitatively inherited. Tomato (*Solanum lycopersicum* L.) is an important model plant species for understanding fleshy fruit development and morphology [8]. To date, six genes affecting fruit weight have been cloned, including *FW2.2* [9], *FW3.2* [10], *FW11.3* [11], *FAS* [12], *LC* [13], and *Globe* [14]. Among them, *FW2.2* (CNR) and *FW3.2* (SlKLUH) increases in cell layers are achieved by changing cell division rates and/or duration in the pericarp of the fruit [9,10]. *FW2.2* is a negative regulator and is mainly expressed in the ovary at anthesis [9], while *FW3.2* impacts cell layers mainly in the pericarp of the developing fruit instead of the ovary [10]. *FW11.3* is a cell size regulator (CSR) [11]. *FAS* and *LC* affect fruit weight by affecting the number of chambers [12,13]. *GLOBE* is a newly discovered gene affecting tomato fruit weight [14]; it mainly affects the shape of the fruit. Additionally, transcription factors such as MADS-box and MIXTA-like MYB and DREB3 also play an important role in tomato fruit development [15]. Compared with tomato, there are few studies about fruit weight in pepper.

Fruit weight in pepper is governed by a number of quantitative trait loci (QTL) that are related to fruit length, fruit width, locule number, flesh thickness, and so on. To date, many QTL that affect the fruit weight in pepper have been found, mainly by QTL mapping studies, BSA-seq, and genome-wide association analyses. Previously, researchers searched for the QTL associated with fruit weight by traditional genetic map-based QTL mapping [16,17,18,19]. By constructing a genetic map of 1740 cM, five QTL, *fw2.1*, *fw3.1*, *fw3.2*, *fw4.1*, and *fw8.1*, were detected for fruit weight [17]. A major QTL, *fw2.1*, was identified on chromosome 2, which explained 62% of the trait variation, using *C. chinense* and *C. frutescens* introgression lines (ILs) of chromosomes 2 and 4 [18]. Rao et al. [19] constructed an interspecific genetic map from a cross between the *C. annuum* cultivated species and the wild *C. frutescens* species, and eight fruit weight QTL, which may be orthologous to tomato fruit weight QTL, were identified in the study.

In addition to traditional genetic map-based QTL mapping, with the publication of the pepper genome [20,21], sequencing-based mapping approaches, such as QTL-seq and genome-wide selection strategies (GWAS), promote a higher resolution and faster mapping of genomic regions for fruit weight in pepper [22]. Using the natural populations of 94 materials, 16 SNPs strongly associated with pepper fruit weight were obtained using a GWAS analysis in two years, and 7 SNPs are located in known fruit weight controlling genes. Among them, two nonsynonymous SNPs, S6_227195619 and S10_229225552, were located in chloroplastic FAF-like protein and cell division control protein 45 (CDC45), respectively, which related to biological processes involving cell division and meristem organization [23]. A GWAS analysis of 350 core accessions revealed seven QTL associated with fruit weight, and seven possible candidate genes were further identified. Among them, a homolog of BEL1-LIKE HOMEODOMAIN PROTEIN 2 (BLH2) underlying the QTL *FWe-P2.4*, detected with the strongest marker–FWe association, may thus affect fruit weight [24]. In addition to genome sequencing analysis approaches, transcriptome- and proteome-wide association methods have also been applied to discover the QTL for fruit weight in pepper. Five QTL located on LG2, LG4, LG6, and LG7 and 42 genes related to fruit weight were identified, respectively, using transcriptome- and proteome-wide association in a recombinant inbred line population [25]. Recently, according to a transcriptomic analysis, it has been determined that boron transporter 4, the auxin signal transduction pathway genes (CaAUX1, CaAUX/IAA, CaGH3, CaSAUR), and transcription factors (CaMADS3, CaAGL8, CaATHB13, CaATHB-40) may be involved in the growth and development of pepper fruit [26].

Although many QTL of fruit weight have been reported in pepper, and some candidate genes were predicted, the underlying genetic mechanism of this trait is almost unknown, which restricts the genetic improvement in fruit weight in pepper breeding. In this study, we aimed to identify and analyze fruit weight-related QTL in an intraspecific *C. annuum* cross between the sweet blocky cultivated pepper accession “Qiemen” and the bird wild accession “129−1” (*C. annuum* var. *glatriusculum*). Two major QTL, *qFW2.1* and *qFW3.1*, which are related to fruit weight, were identified and fine-mapped on chromosomes 2 and 3, and three candidate genes were further screened using RNA-seq and qRT-PCR. The result lays a theoretical foundation for the cloning of *qFW2.1* and *qFW3.1*, and also provides an important basis for further understanding of pepper fruit development biology and genetic improvement.

## 2. Materials and Methods

### 2.1. Plant Materials

In this study, the blocky-type cultivated pepper “Qiemen” (*C. annuum* L., weight per fruit about 125.00 g) and the wild pepper species “129−1” (*C. annuum* var. *glatriusculum*, wolfberry fruit, weight per fruit about 0.15 g) were used as parents. The fruit of wild pepper 129−1 is small (about 1.00 cm in length), erect, red-colored, pungent (hot), deciduous (falls off the plant when ripe), and soft-fleshed. We used “Qiemen” as the female parent and “129−1” as the male parent to develop F_1_ and F_2_ populations. All the peppers were provided by the pepper research group of the Institute of Vegetables and Flowers, Chinese Academy of Agricultural Sciences. In the autumn of 2021, two F_2_ populations, which were named 2021SY47 and 2022SG136, derived from a cross between Qiemen and 129−1, were grown in a greenhouse with normal water and fertilizer management in Shunyi District, Beijing (40°17′ N, 116°87′ E), and Shouguang County of Shandong Province (36°91′ N, 118°86′ E), China, respectively. These two F_2_ populations, which comprised 279 and 367 individuals, respectively, were used for a QTL-seq analysis and primary mapping. In addition, F_1_ was backcrossed by the recurrent parent Qiemen to construct a BC_2_ population. In the autumn of 2022, a total of 29 BC_2_ individuals with different heterozygous segments for the target QTL interval (*qFW2.1* or *qFW3.1*) were selected to generate BC_2_F_2_ and BC_2_F_2:3_ heterozygous inbred lines (HIFs) for verifying and a further fine-mapping analysis of QTL *qFW2.1* and *qFW3.1*. All 29 BC_2_ individuals were screened by the background marker to make true the genotypes of other possible fruit weight QTL regions fixed for the parent “Qiemen” allele. A total of 10–15 plants with homozygous parental genotypes at the exchange site were selected from HIFs and planted in a plastic greenhouse for measuring the fruit weight characteristics. Each plant was weighed with 5–6 uniform mature green fruits, and the average value was calculated. And four characteristics including fruit weight with stalk (FWs), fruit weight without stalk (FW), fruit length (FL), and fruit diameter (FD) were selected to be measured. The fruit weight data and correlations between the four fruit weight characteristics were analyzed using IBM SPSS Statistics 23 (SPSS Inc., Chicago, IL, USA) software.

### 2.2. DNA Extraction, PCR Amplification, and Detection

The genomic DNA of the pepper was extracted using the CTAB method [27], when two leaves were fully expanded and one new leaf began to appear. The integrity and purity of the DNA samples were determined using 1% agarose gel electrophoresis, and the concentration of DNA was measured using NanoDrop (Thermo Fisher Scientific, Wilmington, DE, USA). The final DNA concentrations were diluted to 50–100 ng/µL with ddH_2_O as the working solution. For PCR, a 1 µL DNA sample was taken and put into a reaction volume of 10 µL using gene-specific primers. The amplification of the markers was conducted using the following profile: 94 °C for 4 min; 32 cycles of 15 s at 94 °C, 15 s at optimal annealing temperature, and 30 s at 72 °C; and 72 °C for 5 min. The PCR products were separated on 8% polyacrylamide gel.

### 2.3. QTL-Seq Analysis

According to the fruit weight data of the 2021SY47 population, 28–30 individual plants with extreme fruit weight were selected to construct two sequencing mixed pools, the heavy fruit pool (H-pool) and light fruit pool (L-pool). The two parents, the H-pool and the L-pool, were sequenced on an Illumina HiSeq 4000 system in the PE150 mode [28]. The high-quality data were obtained after removing the low-quality reads (Q < 20), adapter sequences, N > 10% reads, and too-short reads (<20 bp) by the software of Cutadapt (version 1.17) and Trimmomatic. The clean reads were then mapped to the Zunla-1 v2.0 reference enome [21] using BWA (version 0.7.17) software [29]. The variation detection (including InDels and SNPs) was conducted by using the software SAMtools (version 1.17) [30] and GATK [31]. Before association analyses were undertaken, SNPs were filtered. The filtering criteria were as follows: SNPs with multiple genotypes were filtered; SNPs with read supports < 4 were filtered; SNPs with consistent genotypes in two pools were filtered. After filtering the InDels and SNPs, the homozygous and polymorphic loci were retained for a follow-up analysis. The SNP index and Δ SNP index were calculated using the BSA-seq policy [22]. The SNP index and Δ SNP index were calculated with a 1 Mb interval and 50 kb sliding window to identify genomic regions for Zunla-1 v2.0. The (SNP index) diagram was drawn, and 1000 permutation tests were carried out. The 95% and 99% confidence levels were selected as the thresholds for screening possible genomic regions associated with the fruit weight trait.

### 2.4. QTL Mapping with InDel Marker

According to the preliminary mapping results of the QTL-seq analysis, the F_2_ population was further used to map and verify the initial location area. Sequences of the candidate regions associated with the fruit weight trait, which were identified by QTL-Seq, were downloaded from the reference genome, and the amplification primers were designed using BatchPrimer3 (https://primer3.ut.ee/, accessed on 3 March 2024) Inpult online software at Primer3 Input [32]. Polymorphism verification was carried out between the parents, and polymorphic and specific markers were selected to detect the F_2_ population genotypes. QTL IciMapping 4.1 was used to construct the local genetic linkage map of chromosomes [33], and the genetic linkage relationship between the polymorphic markers was analyzed. Using all phenotyping data and genetic maps, the QTL analysis was conducted in the F_2_ population using the Inclusive Composite Interval Mapping of Additive (ICIM-ADD) module within QTL IciMapping 4.1. The LOD threshold was determined using 1000 permutations with a 5% probability for each chromosome and each trait. Genomic regions with LOD values greater than 3.0 were considered to have QTL related to fruit weight. We constructed fine positioning using HIFs derived from the recombinants of BC_2_. If there was a significant difference in fruit weight between the two groups, which contained homozygous parental segments at the exchange site, respectively, this interval was considered to contain the QTL related to fruit weight.

### 2.5. Transcriptome Analysis

Three fruits were randomly collected from two parent individuals at 1, 15, 25, and 35 DAP (days after pollination). RNA was extracted from the pericarps of the fruit, and three biological replicates represented each fruit development stage. The quality and quantity of total RNA were assessed by a NanoDrop2000 spectrophotometer (Thermo Fisher Scientific Inc., Wilmington, DE, USA). A total of 24 RNA samples were used to construct the cDNA library and resequenced by the Illumina HiSeq-PE150 sequencing platform at Beijing Biomarker Technologies Corporation (Beijing, China, http://www.biomarker.com.cn, accessed on 3 March 2024). The raw data were processed for quality control to obtain clean data using fastp (version 0.19.7) software. After quality control, clean reads were mapped to the Zunla-1_v2.0 reference genome using HISAT2 (version 2.2.0) software. The FPKM of each gene was calculated based on the length of the gene, and the read count mapped to this gene for a differential expression analysis was processed using DESeq2 [34]. DEGs were defined as genes with an FDR ≤ 0.01 and |log2 (fold change)| ≥ 1.

### 2.6. Verification of Gene Expression Pattern Using qRT-PCR

The functional annotation of the predicted genes in the region of *qFW2.1* and *qFW3.1* was found by BLASTN pepper full-length cDNA searching against databases, including the Sol Genomics Network (SGN, http://solgenomics.net, accessed on 3 March 2024), Unigene, ESTs, and the National Center for Biotechnology Information (NCBI, http://www.ncbi.nlm.nih.gov, accessed on 3 March 2024).

A total of nineteen candidate genes for *qFW2.1* and *qFW3.1*, detected using RNA-seq combining with a variation analysis, were selected for validation by qRT-PCR. Total RNA was extracted from 129−1 and Qiemen at 1 DAP, 11 DAP, 25 DAP, and 35 DAP with VeZol Reagent (Vazyme, Nanjing, China). The cDNA synthesis and PCR amplification were performed using the HiScript IV 1st Strand cDNA Synthesis Kit and ChamQ Blue Universal SYBR qPCR Master Mix (Vazyme, Nanjing, China) according to the manufacturer’s instructions. The 10 µL reaction system PCR reaction was performed using LC480 real-time PCR (Roche Diagnostics, Rotkreuz, Switzerland). The double-delta CT (2^−ΔΔCT^) method was conducted to calculate the relative gene expression levels. Actin [35] was applied as an internal control. Three biological replicates were used per development stage, and average transcript levels of each sample were calculated from three technical replicates. The qRT-PCR primer sequences are listed in Table S11.

## 3. Results

### 3.1. Phenotypic Analysis of F_2_

**Figure 1:**
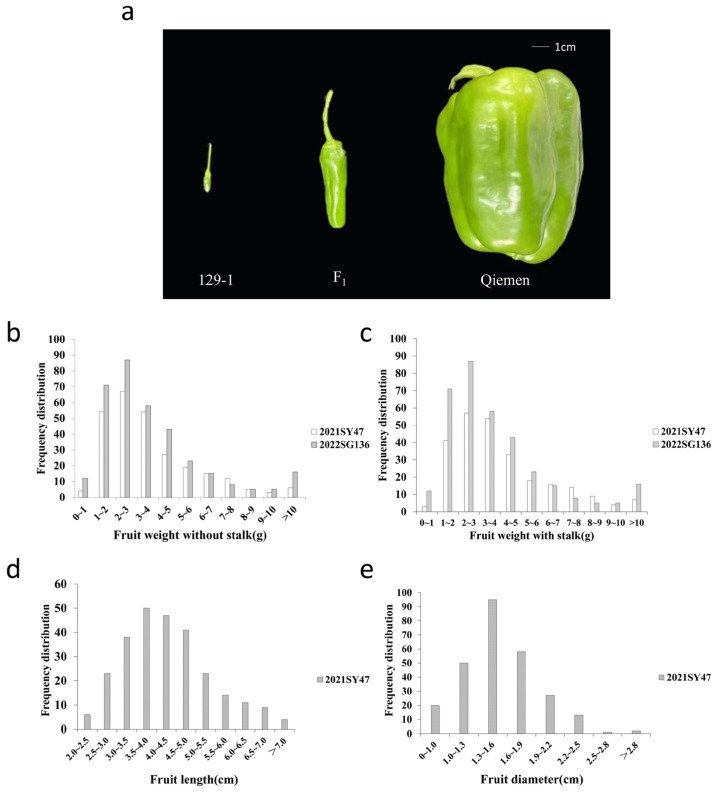
Frequency distribution of F_2_ (Qiemen × 129−1). (**a**): Fruit of parents and F_1_ fruit. (**b**): Frequency distribution per fruit weight without stalk removed in F_2_ population (Qiemen × 129−1). (**c**): Frequency distribution per fruit weight with stem removed in F_2_ population (Qiemen × 129−1). (**d**): Frequency distribution of fruit length removed in F_2_ population (Qiemen × 129−1). (**e**): Frequency distribution of fruit diameter removed in F_2_ population (Qiemen × 129−1).

The mean FW for the heavy fruit parent, Qiemen, and light fruit parent, 129−1, was 125.00 g and 0.15 g, respectively (Figure 1a, Table S1). The F_1_ hybrid had an average FW of 5.07 g and fruit shape appeared to be the finger type (Figure 1a). As shown in Figure 1, in the F_2_ populations, the FW, FWs, and Fl are skewed towards 129−1. The Fd of F_2_ is in the middle of the parental lines. The FW of the 2021SY47 population ranged from 0.67 g to 21.58 g, with an average of 3.72 g; the FWs of the 2021SY47 population ranged from 0.82 g to 22.67 g, with an average of 3.99 g; the fruit length of the 2021SY47 ranged from 2.06 cm to 7.87 cm, with an average of 4.29 cm; and the fruit diameter of the 2021SY47 ranged from 0.83 cm to 3.53 cm, with an average of 1.54 cm. The fruit weight of the 2022SG136 population ranged from 0.55 g to 20.99 g, with an average of 3.85 g. The frequency distributions of fruit weight traits in the two F_2_ populations conformed to an approximately continuous normal distribution, which indicates that these traits are quantitative traits controlled by multiple genes (Figure 1). The FW was significantly positively correlated with the FWs (*r* = 0.999, *p* < 0.01), FL (*r* = 0.826, *p* < 0.01), and FD (*r* = 0.826, *p* < 0.01). This suggests that fruit weight may be affected by both fruit length and fruit diameter. Thus, the FW was used as the main phenotypic data for genetic mapping in the following study due to strong correlation among these four traits.

### 3.2. Two Major QTL Were Identified by Using QTL-Seq

**Figure 2:**
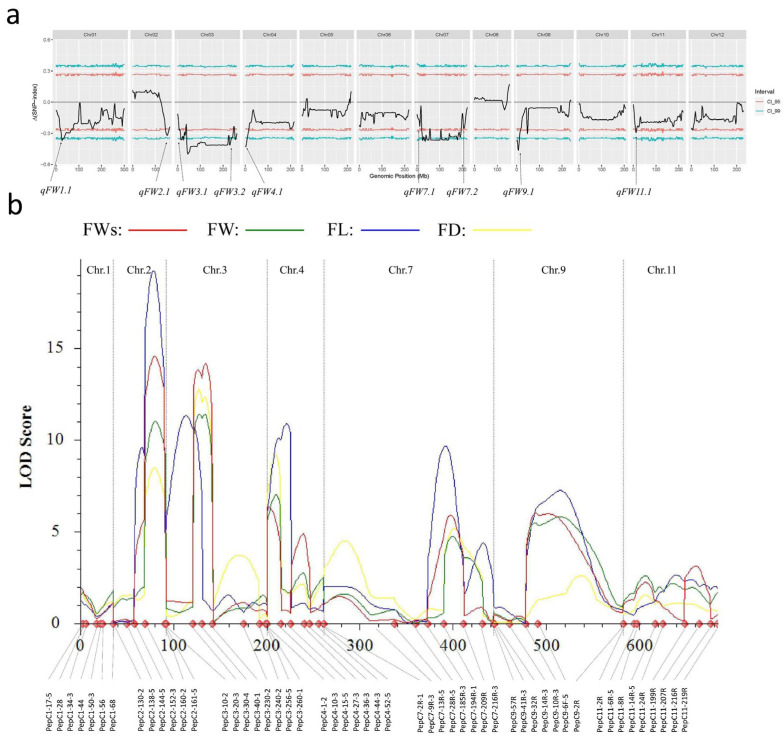
The QTL analysis for fruit weight using 2021SY47 populations. (**a**) The Δ (SNP index) Manhattan graphs of 12 chromosomes. The region indicated by the arrow indicates that the region may contain QTL related to fruit weight, the blue curve indicates the 99% confidence interval, the red curve indicates the 95% confidence interval, and the black line indicates Δ (SNP index). (**b**) Linkage-based QTL mapping of 9 candidate QTL. The genetic linkage analysis of the 2021SY47 population was performed using 52 InDel markers; five QTL related to FW were detected in 2021SY47 populations, including *qFW2.1*, *qFW3.1*, *qFW4.1*, *qFW7.2*, and *qFW9.1*. The *qFW2.1* and *qFW3.1* were major QTL.

After whole-genome resequencing, 516.35, 542.04, 867.92, and 827.34 million clean reads were generated from Qiemen, 129−1, H-pool, and L-pool, respectively (Table S2). After quality control, 95.94–96.50% of the read pairs in the four libraries were uniquely mapped to the Zunla-1 v2.0 reference genome. The depths of Qiemen, 129−1, H-pool, and L-pool are 27.99×, 29.21×, 46.82×, and 44.73×, respectively. A total of 24,964,557 high-quality SNPs and 1,970,887 InDels were identified between the two parental lines. To identify the major QTL related to fruit weight, the SNP index was calculated for each bulk by aligning the sequences to the reference genome. By combining the information of the SNP index in the H-pool and L-pool, a ∆ (SNP index) was calculated and plotted against the genome positions. A total of nine candidate QTL regions related to fruit weight were identified with a statistical confidence of *p* < 0.05 (Figure 2a). These were designated as *qFW1.1*, *qFW2.1*, *qFW3.1*, *qFW3.2*, *qFW4.1*, *qFW7.1*, *qFW7.2*, *qFW9.1*, and *qFW11.1*, respectively. In order to validate the result of QTL-seq, a total of 184 InDel markers were developed in the nine candidate regions and screened the polymorphism between the parents. A total of 52 InDel markers (Table S3) with clear bands and polymorphisms were selected to construct a linkage map using the QTL IciMapping 4.0 software. Two major QTL for fruit weight, *qFW2.1* and *qFW3.1*, were verified by the ICIM mapping analysis in 2021SY47 populations, with LOD thresholds of 11.02 and 11.39, respectively. The *qFW2.1* was delimited by two InDel markers, PepC2-144-5 and PepC2-160-2, which were physically located in the region of 143.83–160.36 Mb on chromosome 2 (Figure 2b, Table S4). *qFW3.1* was delimited by two InDel markers, PepC3-10-2 and PepC3-40-1, which were physically located in the region of 9.80–40.82 Mb on chromosome 3 (Figure 2b, Table S4). In addition, we also located other traits, and the QTL of weight with the stalk, length, and diameter of fruit in 2021SY47 all coincided with the QTL of weight without stalk.

At the same time, markers of the major effect interval were used for the genetic linkage analysis in the 2022SG136 population. In the 2022SG136 population, *qFW2.1* was also located to markers PepC2-144-6 and PepC2-160-2 on chromosome 2, with an LOD value of 7.36, and the maximum phenotypic variation explained 7.17%. *qFW3.1* was also located between marker PepC3-10-2 on chromosome 3 and marker PepC3-40-1 with an LOD value of 10.46, and the maximum phenotypic variation explained 15.50%. The *qFW2.1* and *qFW3.1* had the highest explanation of phenotypic variation, and they were stable in the two populations. The fruit weight without stalk was the main character studied.

### 3.3. Genetic Linkage Analysis Narrowed qFW2.1 and qFW3.1 Candidate Region

In order to narrow the candidate interval of *qFW2.1* and *qFW3.1*, 36 InDel markers within the candidate intervals were developed on chromosome 2 and 3, and 15 with a steady and clear spectrum were selected for their polymorphism in the two parents. The two major fruit weight QTL, *qFW2.1* and *qFW3.1*, were further narrowed using the linkage analysis. The *qFW2.1* was in the region between markers PepC2-152-3 and PepC2-156-1 with a physical distance of approximately 4.46 Mb on chromosome 2. The *qFW3.1* was in the region between markers PepC3-12-1 and PepC3-18-1 with a physical distance of approximately 5.66 Mb on chromosome 3 (Table 1).

**Figure 3:**
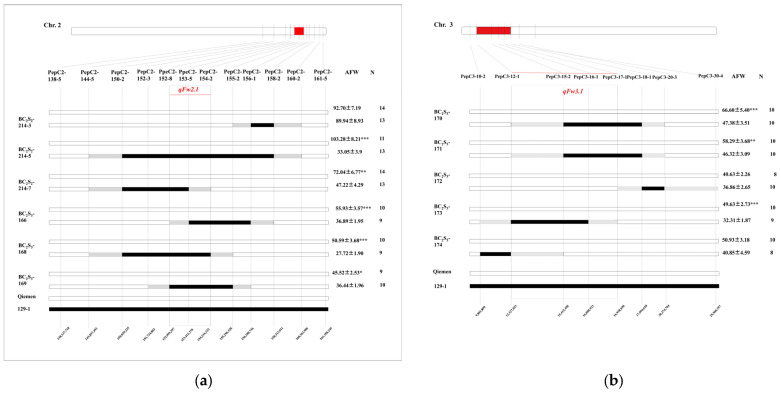
Dissection and validation of *qFW2.1* and *qFW3.1* region using HIFs. (**a**) Progeny tests of homozygous recombinants localized *qFW2.1* to region flanked by markers PepC2-152-8 and PepC2-154-2 and comparison of mean of fruit weight between two homozygous individuals (129−1 allele and Qiemen allele) within each family. Groups BC_2_S_2_-214-3, BC_2_S_2_-214-5, BC_2_S_2_-214-7, BC_2_S_3_-166, BC_2_S_3_-168, and BC_2_S_3_-168 are different kinds of recombinant individuals derived from plants that are heterozygous between PepC2-144-5 and PepC2-160-2. (**b**): Progeny tests of homozygous recombinants localized *qFW3.1* to region flanked by markers PepC3-12-1 and PepC3-17-1 and comparison of mean of fruit weight between two homozygous individuals (129−1 allele and Qiemen allele) within each family. Groups BC_2_S_3_-170, BC_2_S_3_-172, BC_2_S_3_-173, and BC_2_S_3_-174 are different kinds of recombinant individuals derived from plants that are heterozygous between PepC3-10-2 and PepC3-20-3. AFW: average of fruit weight; N: numerals refer to number of homozygous individuals. Asterisks indicate significance determined by Student’s *t*-test for each family. *: *p* < 0.05; **: *p* < 0.01; ***: *p* < 0.001.

To better define the fruit weight region, a total of 508 individuals of the BC_2_ population were genotyped to screen the recombinants, which were recombinant in the *qFW2.1* or *qFW3.1* regions. The PepC2-144-5~PepC2-158-2 interval (narrowed for *qFW2.1*) or PepC3-10-2~PepC3-20-3 interval (narrowed for *qFW3.1*) were heterozygous while other possible regions (such as *qFW4.1* and *qFW7.2* loci) were homozygous for the Qiemen alleles. According to their marker genotypes, six recombinants located at the *qFW2.1* region were identified in the BC_2_ population. All homozygous recombinants at the *qFW2.1* region were further classified into four groups based on their breakpoints. When comparing the fruit weight of the non-recombinants with the recombinants in each family, a highly significant (*p* < 0.001) difference in mean fruit weight for BC_2_S_2_-214-7 was observed and an increase of 57.61% in fruit weight. These results indicated that the *qFW2.1* locus was located in the PepC2-152-3~PepC2-154-2 interval on chromosome 2, with a physical distance of approximately 2.50 Mb (Figure 3a, Table S5). There was no recombinant between PepC3-12-1 and PepC3-18-1.

To further delimit the genomic regions of *qFW2.1* and *qFW3.1*, the BC_2_S_2_ population containing 864 individuals was genotyped with flanking markers and nine recombinants were identified corresponding to an interval of BC_2_S_3_-165, BC_2_S_3_-166, BC_2_S_3_-168, and BC_2_S_3_-169 (*qFW2.1*) and BC_2_S_3_-170, BC_2_S_3_-171, BC_2_S_3_-172, BC_2_S_3_-173, and BC_2_S_3_-174 (*qFW3.1*), respectively. For the QTL region of *qFW2.1*, the homozygous recombinants were divided into three groups. For groups BC_2_S_3_-165, BC_2_S_3_-166, BC_2_S_3_-168, and BC_2_S_3_-169, the mean fruit weight of the recombinants was significantly higher (*p* < 0.01) than that of the non-recombinants. These results indicated that the *qFW2.1* locus was located in the PepC2-152-3~PepC2-154-2 interval on chromosome 2, with a physical distance of approximately 1.22 Mb. For the QTL region of *qFW3.1*, the homozygous recombinants were divided into four groups. For groups BC_2_S_3_-170, BC_2_S_3_-171, and BC_2_S_3_-173, the mean fruit weight of the recombinants was significantly higher (*p* < 0.01) than that of the non-recombinants. These results indicated that the *qFW2.1* locus was located in the PepC3-12-1~PepC3-17-1 interval on chromosome 3, with a physical distance of approximately 4.61 Mb (Figure 3, Tables S5 and S6).

### 3.4. Differential Gene Expression Analysis between the Two Parents

To investigate the candidate genes associated with the fruit weight of pepper, an RNA-seq analysis was performed. The respective numbers of DEGs in the “Qiemen” vs. “129−1” comparisons were 9834, 7451, 7762, and 8554 at 1 DAP, 15 DAP, 25 DAP, and 35 DAP (Figure S1). A total of 1776 DEGs were overlapped in all four periods. GO enrichment and KEGG pathway analyses of the DEGs were conducted. At 1 DAP, the two most significantly enriched GO terms were the MCM complex and photosystem II oxygen evolving complex in the cellular component category. DNA helicase activity and ribonuclease T2 activity were the two most significantly enriched GO terms in the molecular function category. The two most significantly enriched GO terms were protein ubiquitination and DNA replication in the biological process category (Figure S2). At 15 DAP, the two most significantly enriched GO terms were photosystem I and the integral component of the membrane in the cellular component category. Xyloglucan/xyloglucosyl transferase activity and peptidase inhibitor activity were the most significantly enriched GO terms in the molecular function category. The two most significantly enriched GO terms were peptidase inhibitor activity and photosynthesis and light harvesting in the biological process category (Figure S3). At 25 DAP, the two most significantly enriched GO terms were photosystem I and photosystem II in the cellular component category. Trehalose-phosphatase activity and trehalose-phosphatase activity were the most significantly enriched GO terms in the molecular function category. The two most significantly enriched GO terms were photosynthesis, light harvesting and photosynthesis, and light harvesting in the biological process category (Figure S4). At 35 DAP, the two most significantly enriched GO terms were nucleosome and nucleosome in the cellular component category. Microtubule binding and monooxygenase activity were the most significantly enriched GO terms in the molecular function category (Figure S5). The two most significantly enriched GO terms were monooxygenase activity and the carbohydrate metabolic process in the biological process category.

Additionally, the KEGG database was used to analyze the DEGs. The top 20 enriched KEGG pathways are displayed in Figures S6–S9. These differential genes were significantly enriched into biosynthesis of various secondary metabolites, plant hormone signal transduction, photosynthesis, and other pathways (Figures S6–S9). Because fruit is regulated by plant hormones such as gibberellin, auxin, and cytokinin during development, we focused on genes that are enriched in “plant hormone signal transduction”. A total of 184, 166, 151, and 172 DEGs at 1, 15, 25, and 35 DAP were clustered in plant hormone signal transduction pathways. The KEGG pathway enrichment analysis provided an important clue for identifying candidate genes. *Capana02g002989*, *Capana02g003021*, *Capana03g000857*, and *Capana03g001031* are enriched on this pathway.

### 3.5. Prediction and Analysis of Candidate Genes for the Fruit Weight

Among the genes that were significantly differentially expressed between the “Qiemen” and “129−1”, we focused on genes located within the QTL to identify candidate genes associated with fruit weight. A total of 47 genes were discovered in the regions where two QTL overlapped on chromosome 2. A total of 86 genes were discovered in the regions where two QTL overlapped on chromosome 3 (Table S9).

**Figure 4:**
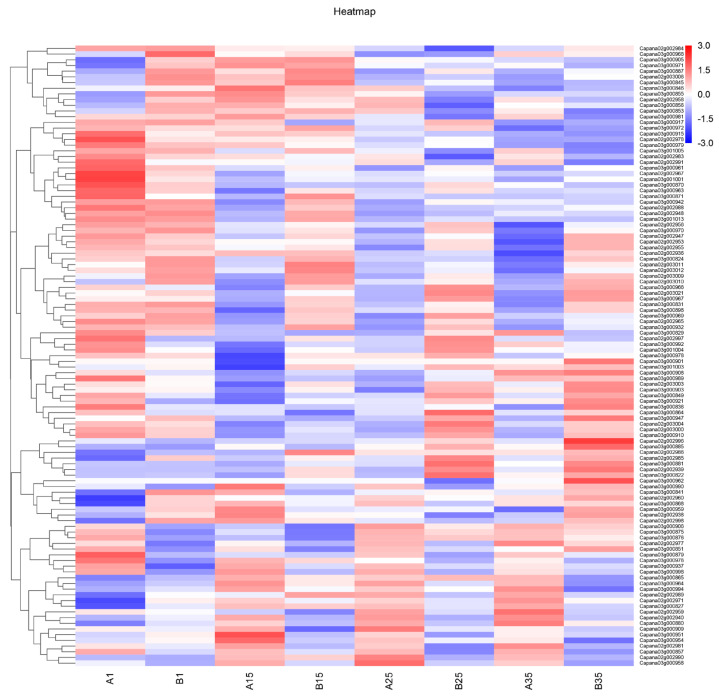
A heat map of differentially expressed genes of *qFW2.1* and *qFW3.1*. A1: The expression at 1 DAP of Qiemen; A15: The expression at 15 DAP of Qiemen; A25: The expression at 25 DAP of Qiemen; A 35: The expression at 35 DAP of Qiemen; B1: The expression at 1 DAP of 129−1; B15: The expression at 15 DAP of 129−1; B25: The expression at 25 DAP of 129−1; B35: The expression at 35 DAP of 129−1.

Based on the results of resequencing data, 24 genes with no sequence difference between “Qiemen” and “129−1” were further excluded (Table S10). The expression of all the remaining genes is shown in Figure 4.

Combined with the gene annotation of the Zunla-1_v2.0 reference genome and the search for homologous genes, 19 genes that were most likely related to fruit weight were selected for verification by the qRT-PCR analysis. The presumed functions of these 19 genes are in Table 2. Both the qRT-PCR and RNA-seq analysis generally showed similar expression patterns of the up- and down-regulation of these 19 DEGs. Among them, three genes, *Capana02g002938*, *Capana02g003021*, and *Capana03g000903*, showed the most significant expression level difference between the transcriptomes of the parent “Qiemen” and “129−1”. *Capana02g002938*, located on Chr02: 153038618–153038618, encodes growth-regulating factor 2-like (GRF); *Capana02g002938* exhibited significantly higher expression levels at 11 DAP and 35 DAP in “129−1” than in “Qiemen”. *Capana02g003021*, located on Chr02: 154187708–154190076, encodes GRETCHEN HAGEN3-type (GH3) protein; *Capana02g003021* exhibited significantly higher expression levels at 25 DAP and 35 DAP in “129−1” than in “Qiemen”. *Capana03g000903*, located on Chr03: 14554077–14555759, encodes C78A4_PINRA Cytochrome P450; *Capana03g000903* exhibited significantly higher expression levels at 11 DAP in “129−1” than in “Qiemen”.

**Figure 5:**
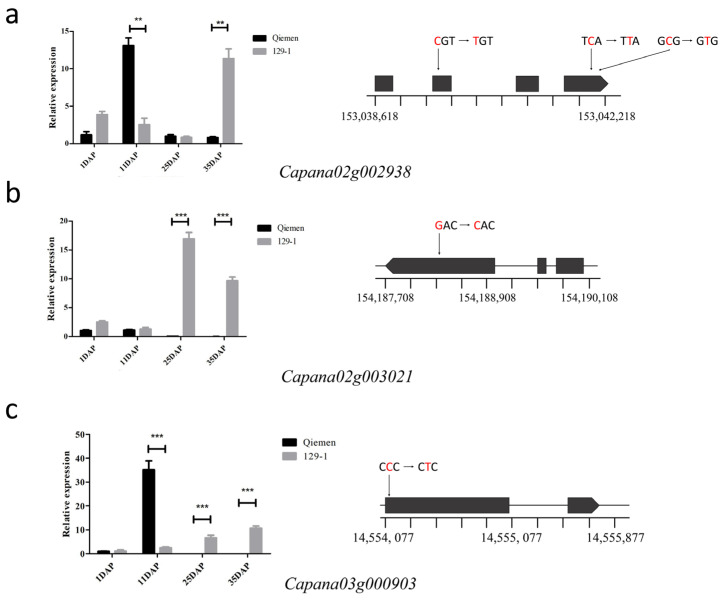
Validation and expression analysis of selected genes using qRT-PCR. (**a**) Sequence difference of *Capana02g002938* in Qiemen and 129−1 and expression difference in different developmental stages of fruit; (**b**) sequence difference of *Capana02g003021* in Qiemen and 129−1 and expression difference in different developmental stages of fruit; (**c**) sequence difference of *Capana03g000903* in Qiemen and 129−1 and expression difference in different developmental stages of fruit. **: *p* < 0.01; ***: *p* < 0.001.

The sequence comparison of the gene-coding regions of *Capana02g002938*, *Capana02g003021*, and *Capana03g000903* between “129−1” and “Qiemen” revealed five nonsynonymous SNPs in exons, respectively (Figure 5).

## 4. Discussion

Pepper is an important vegetable crop that is widely grown around the world. The fruit of pepper can be used for vegetables, food additives, and pigment extraction and has high medicinal and economic value. Up until now, many traits of the pepper fruit, such as fruit color [36], fruit shape [16], fruit weight [17], fruit locule number [37], etc., were extensively studied using QTL methods. Fruit weight is an important characteristic of pepper, which directly affects the yield of pepper. The size and weight of edible organs have been strongly selected as important characteristics during crop domestication [38]. Previous studies have identified many major and minor QTL controlling fruit weight, which was mainly carried out by interspecific crossing of domesticated peppers with wild accessions or intraspecific crossing in domesticated peppers. Many QTL for fruit weight-related traits have been identified in different genetic backgrounds and were mainly distributed on chromosomes 2, 3, 4, 7, and 10 [17,18,19,20]. However, most fruit weight loci were located in large chromosome intervals due to the low density of the genetic map. Additionally, the molecular regulatory mechanism related to fruit weight of *capsicum* is still unclear.

In this study, we used a wild accession of *C. annuum* (129−1) and cultivated accession (Qiemen) as the parents and species and fine-mapped two major QTL, *qFW2.1* and *qFW3.1*, controlling fruit weight in pepper. Based on the physical positions, *qFW2.1* was located in the marker PepC2-152-8~PepC2-154-2 interval on chromosome 2, with a physical position from approximately 153.00 Mbp to 154.22 Mbp. At the same time, the QTL related to the FL and FD were also detected in the region. The physical position of QTL *qFW2.1* identified in this study was close to that of the previous reported QTL, *ftw2.1* [37] and *FWe-P2.4* [24]. However, the physical position of *qFW2.1* (153.00 Mbp to 154.22 Mbp) in our study was not overlapped with the *ftw2.1*, which was shown by Ma et al. [37]. The *ftw2.1* was detected on chromosome 2, which was located between CA514272 (105.98 cM) and GI712 (123.72 cM). The physical interval was approximately 156 Mbp to 165 Mbp on chromosome 2 in the Zunla-1 reference genome. The fruit weight QTL, *FWe-P2.4*, was detected using multi-environment GWAS in a 350 pepper core accessions [24]. *FWe-P2.4* was physically located in Chr02: 160,335,675bp−161,135,675bp in CM334 reference genome version 1.6. CA. *PGAv. 1.6 scaffold836.9* (similar to BLH2) and CA. *PGAv. 1.6 scaffold358.113* (similar to *OVATE*) were the candidate genes for the QTL *FWe-P2.4*. Similarly to the previous study of McLeod et al. [24], the QTL *qFW2.1* identified in our study was corresponded to 161–162 Mb on chromosome 2 in CM334 reference genome version 1.6, but the gene *OVATE* is outside the target position of *qFW2.1. qFW3.1* corresponds to 238–244 Mb on chromosome 3 in the CM334 reference genome [20], which was consistent with a previous reported QTL *FW-3* [39]. Using collinear alignment, the QTL *qFW3.1* was mapped at about 60 Mbp on chromosome 3 in the SL4.0 reference genome [40], which was closed to the cloned tomato fruit weight related gene, *FW3.2* (SlKLUH) [10]. SlKLUH, encoding the ortholog of KLUH, was a P450 enzyme of the CYP78A subfamily. Besides, *CaKLUH*, which was the ortholog of SlKLUH, were identified and found that fruit mass segregated significantly with the large fruited allele of *CaKLUH* from the *C. annuum* parent in a *C. frutescens* × *C. annuum* interspecific RIL population [10]. Additionally, the location region of the two major QTL, especially the *qFW3.1*, is relatively large, which brings difficulty for us to identify candidate genes. Therefore, we plan to use near-isogenic lines to further validate candidate genes, which were predicted in the present study.

To confirm the candidate gene in *qFW2.1* and *qFW3.1*, we screened the results with the related protein annotation and differential expression patterns. Finally, *Capana02g002938* and *Capana02g003021* were found to be candidate genes that may be responsible for the major effects of *qFW2.1*. *Capana02g002938* codes growth-regulating factor 2-like (GRF); GRF is involved in the growth and development of plants, hormonal responses, and so on [41]. The GRF gene in rice has been shown to regulate rice stem growth by mediating gibberellin acid (GA) [42]. Studies have shown that GRF gene expression in meristem tissues is higher than that in mature tissues [43]. In this study, *Capana02g002938* showed differential expression at 11 DAP; three SNPs in the exon region may affect the key sites of the differential expression of this gene.

*Capana02g003021* encodes the GRETCHEN HAGEN3-type (GH3) protein, which is a possible indole-3-acetic acid-amido synthetase. Dynamic changes in indole-3-acetic acid (IAA) cell concentration trigger cell elongation and differentiation [44]. The disruption of the IAA balance by the increased expression of SlGH3.4 or SlGH3.2 results in defective locular and placental tissues in tomato [45]. In this study, *Capana02g003021* was down-regulated in all stages of the fruit development of Qiemen, while it was up-regulated in the middle and late stages of the fruit growth of 129−1. Therefore, we hypothesize that *Capana02g003021* is a fruit growth inhibitor, and an SNP in the exon may be an important cause of this result.

*Capana03g000903* is annotated as a member of the Cytochrome P450 family involved in the proliferation process of fruits. Mainly expressed early in the development of Qiemen fruit, a SNP in the first exon region converts glycine to glutamate. *Capana03g000903* is homologous with tomato *FW3.2* (SlKLUH), which has been proved to be an important gene affecting tomato fruit weight [10,46], which mainly affects fruit weight by controlling cell proliferation in the early stage of fruit development. One SNP in the promoter is highly correlated with fruit weight. Furthermore, in *Arabidopsis*, KLUH regulates cell proliferation by producing novel mobile growth signals that are different from classical plant hormones in a non-cellular autonomic manner, thus controlling organ size in *Arabidopsis thaliana* [47]. This evidence suggested *Capana03g000903* as a candidate gene for *qFW3.1*.

To conclude, our results show that *qFW2.1* and *qFW3.1* are major QTL controlling fruit weight in pepper. We fine-mapped the *qFW2.1* to a 1.22 Mb region located from PepC2-152-8 to PepC2-154-2. *qFW3.1* was located in the region between markers PepC3-12-1 and PepC3-17-1, with a physical distance of approximately 4.61 Mb. Through transcriptomic and qRT-PCR analyses, we conclude that *Capana02g002938* and *Capana02g003021* were the most likely candidate genes of *qFW2.1* and *Capana03g000903* was the most likely candidate gene of *qFW3.1*. This study not only provides a theoretical basis for the cloning of *qFW2.1* and *qFW3.1* of pepper, but also helps us to further understand the molecular mechanism of the fruit weight regulation of pepper.

## Figures and Tables

**Table 1:** Results of F_2_ population initial mapping.

Trait	Population	Chr	LOD	PVE (%)	Left	Right
fruit weight without stalk	2021SY47	2	10.60	12.40	PepC2-152-3	PepC2-156-1
	2022SG136		14.43	14.61	PepC2-150-2	PepC2-154-2
	2021SY47	3	12.87	14.97	PepC3-12-1	PepC3-18-1
	2022SG136		15.37	15.25	PepC3-10-2	PepC3-16-1

**Table 2:** Functional prediction of candidate genes.

Gene ID	Functional Prediction
*Capana02g002936*	AP2-like ethylene-responsive transcription factor ANT
*Capana02g002938*	growth-regulating factor 2-like (GRF)
*Capana02g002971*	adenylyl-sulfate kinase
*Capana02g002977*	mitochondrial outer membrane protein porin 2
*Capana02g002983*	domain-containing protein NPY2
*Capana02g002984*	GCN5-related N-acetyltransferase (GNAT) family protein
*Capana02g002985*	U553_DICDI UPF0553 protein OS
*Capana02g002986*	FMDA_METME Formamidase
*Capana02g002991*	unknown protein
*Capana02g003021*	probable indole-3-acetic acid-amido synthetase GH3.1
*Capana03g000827*	unknown protein
*Capana03g000845*	NDH-DEPENDENT CYCLIC ELECTRON FLOW 1
*Capana03g000846*	UDP-glucose 6-dehydrogenase
*Capana03g000898*	NLI interacting factor (NIF) family protein
*Capana03g000903*	C78A4_PINRA Cytochrome P450
*Capana03g000937*	zinc finger (C2H2 type) family protein
*Capana03g000971*	phosphoglycerate/bisphosphoglycerate mutase family protein
*Capana03g000998*	ARATH Protein PRD1
*Capana03g0001010*	gibberellin-regulated protein 14
*Capana03g0001013*	calmodulin-binding protein

## Data Availability

Data are contained within the article and Supplementary Materials.

## Supplementary Materials

The following supporting information can be downloaded at: https://www.mdpi.com/article/10.3390/genes15081097/s1, Table S1: Investigation of characters of F_2_ population (Qiemen × 129−1); Table S2: Resequencing data in two parents and two bulks; Table S3: The primers used in this study; Table S4: QTL results of fruit weight in F_2_ populations (Qiemen × 129−1); Table S5: Fine mapping result of *qFW2.1* in spring 2023; Table S6: Fine mapping result of *qFW2.1* in autumn 2023; Table S7: Fine mapping result of *qFW3.1*; Table S8: RNA-seq data statistics in four stages of Qiemen and 129−1; Table S9: Genes within the confidence intervals of the two major QTL; Table S10: All the variations in the major effect interval; Table S11: The qRT-PCR primers used in this study; KEGG enrichment analysis of differentially expressed genes 1 day after pollination by both parents; Figure S1. Venn diagram of differentially expressed genes by fruit weight; Figure S2: Go classification enrichment analysis of differentially expressed genes 1 day after pollination by both parents; Figure S3: Go classification enrichment analysis of differentially expressed genes 15 days after pollination by both parents; Figure S4: Go classification enrichment analysis of differentially expressed genes 25 days after pollination by both parents; Figure S5: Go classification enrichment analysis of differentially expressed genes 35 days after pollination by both parents; Figure S6: KEGG enrichment analysis of differentially expressed genes 1 day after pollination by both parents; Figure S7: KEGG enrichment analysis of differentially expressed genes 15 days after pollination by both parents; Figure S8: KEGG enrichment analysis of differentially expressed genes 25 days after pollination by both parents; Figure S9: KEGG enrichment analysis of differentially expressed genes 35 days after pollination by both parents.
