# Supplementary material for: Fine Mapping and Candidate Gene Analysis of Two Major Quantitative Trait Loci, qFW2.1 and qFW3.1, Controlling Fruit Weight in Pepper (Capsicum annuum)
Source: Genes (Basel). 2024 Aug 20;15(8):1097. doi: 10.3390/genes15081097 (PMC11353679; doi:10.3390/genes15081097)
Supplement: Supplementary file 1 [file genes-15-01097-s001.zip › genes-3144266-supplementary_Figures.pdf]

genes

MDPI

Article

Fine Mapping and Candidate Gene Analysis of Two Major Quantitative Trait Loci, *qFW2.1* and *qFW3.1*, Controlling Fruit Weight in Pepper (*Capsicum annuum*)

Supplementary Figures

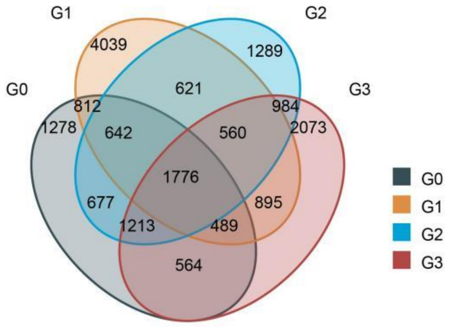

Figure S1. Venn diagram of differentially expressed genes by fruit weight. G0 represents the number of differentially expressed genes 15 DAP between Qiemen and 129-1; G1 represents the number of differentially expressed genes 1 DAP between Qiemen and 129-1; G2 represents the number of differentially expressed genes 25 DAP between Qiemen and 129-1; G3 represents the number of differentially expressed genes 35 DAP between Qiemen and 129-1.

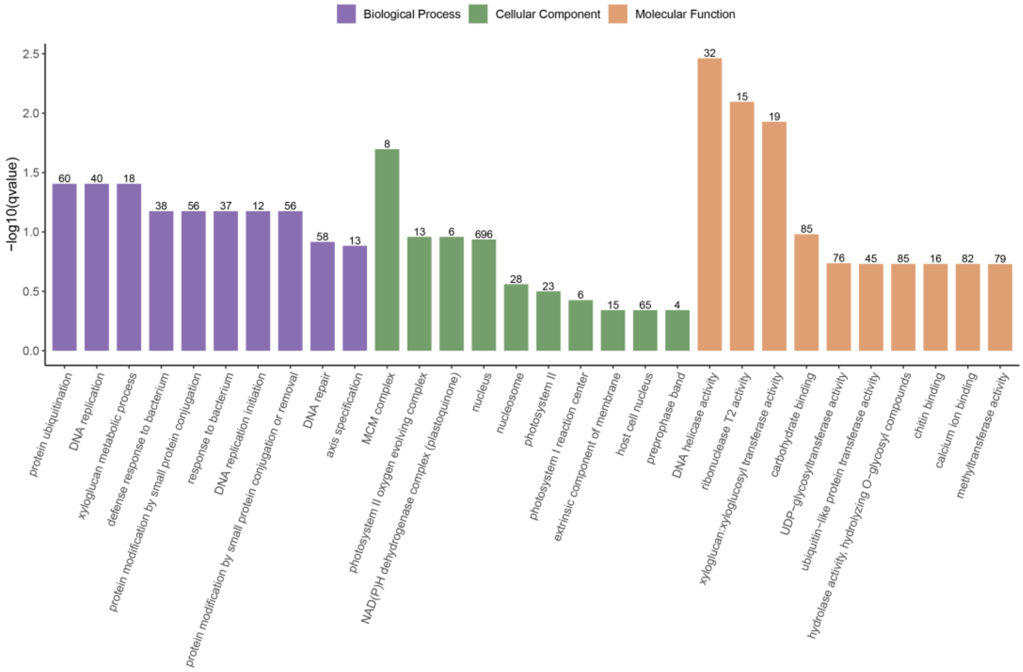

Figure S2. Go classification enrichment analysis of differentially expressed genes 1 day after pollination of between Qiemen and 129-1.

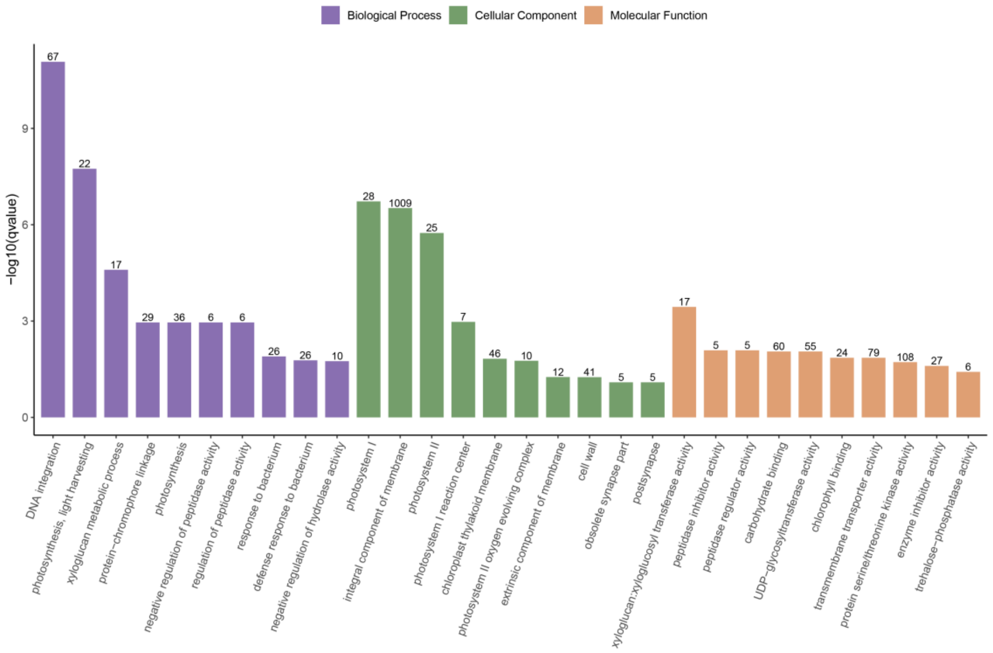

Figure S3. Go classification enrichment analysis of differentially expressed genes 15 day after pollination of between Qiemen and 129-1.

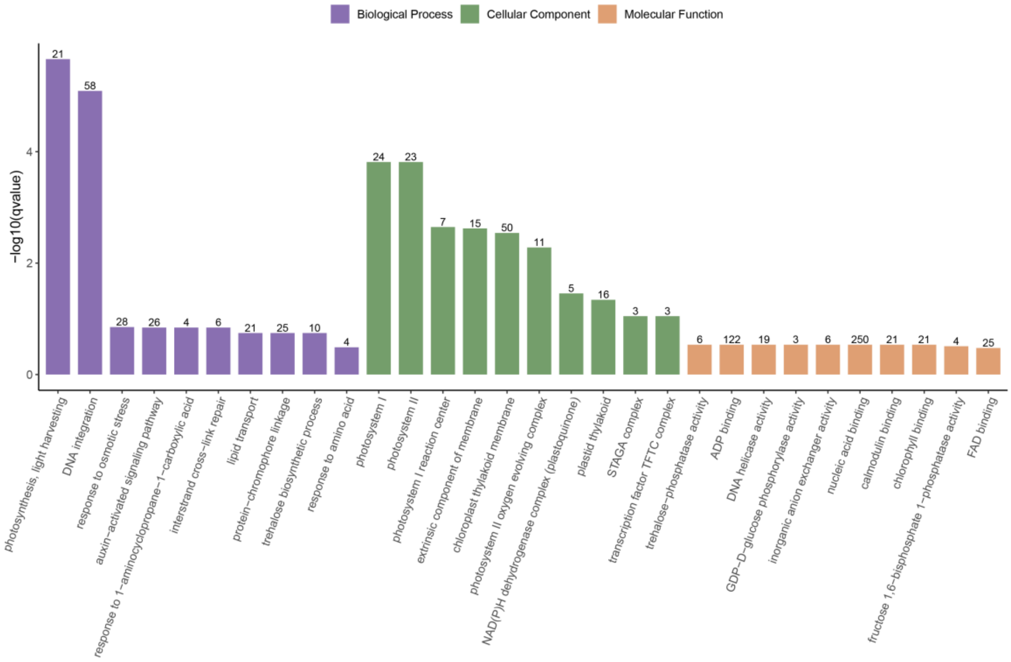

Figure S4. Go classification enrichment analysis of differentially expressed genes 25 day after pollination of between Qiemen and 129-1.

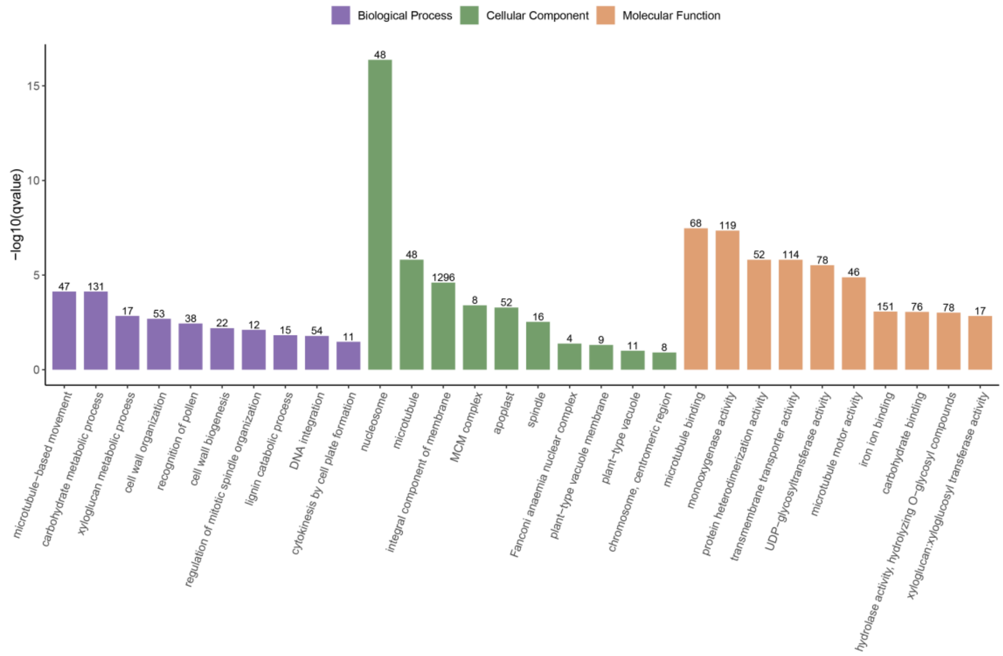

Figure S5. Go classification enrichment analysis of differentially expressed genes 35 day after pollination of between Qiemen and 129-1.

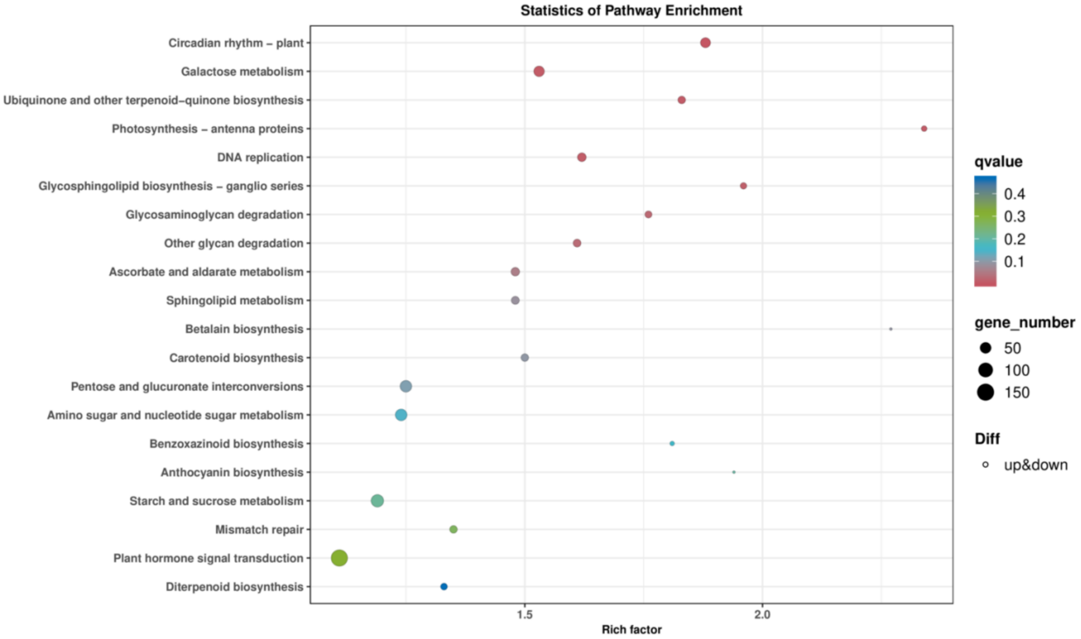

Figure S6. KEGG enrichment analysis of differentially expressed genes 1 day after pollination by between Qiemen and 129-1.

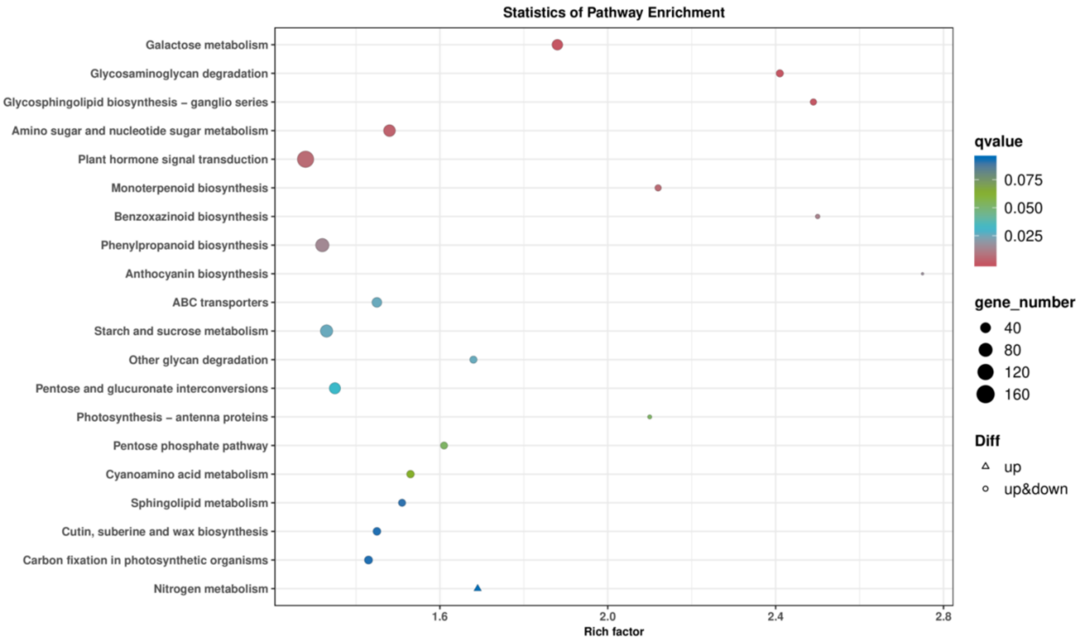

Figure S7. KEGG enrichment analysis of differentially expressed genes 15 day after pollination by between Qiemen and 129-1.

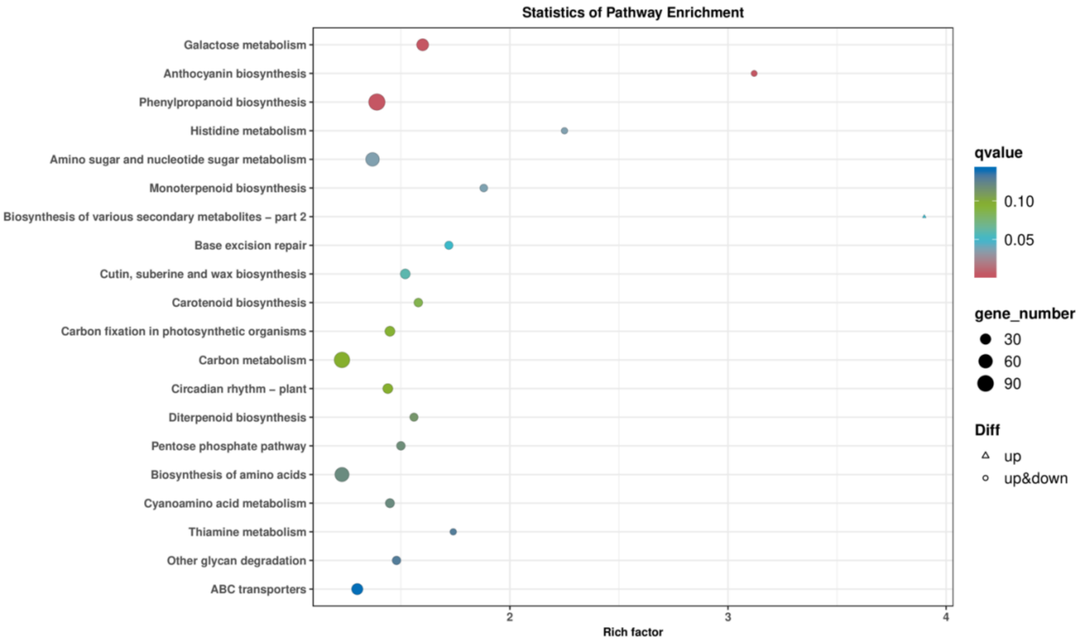

Figure S8. KEGG enrichment analysis of differentially expressed genes 25 day after pollination by between Qiemen and 129-1.

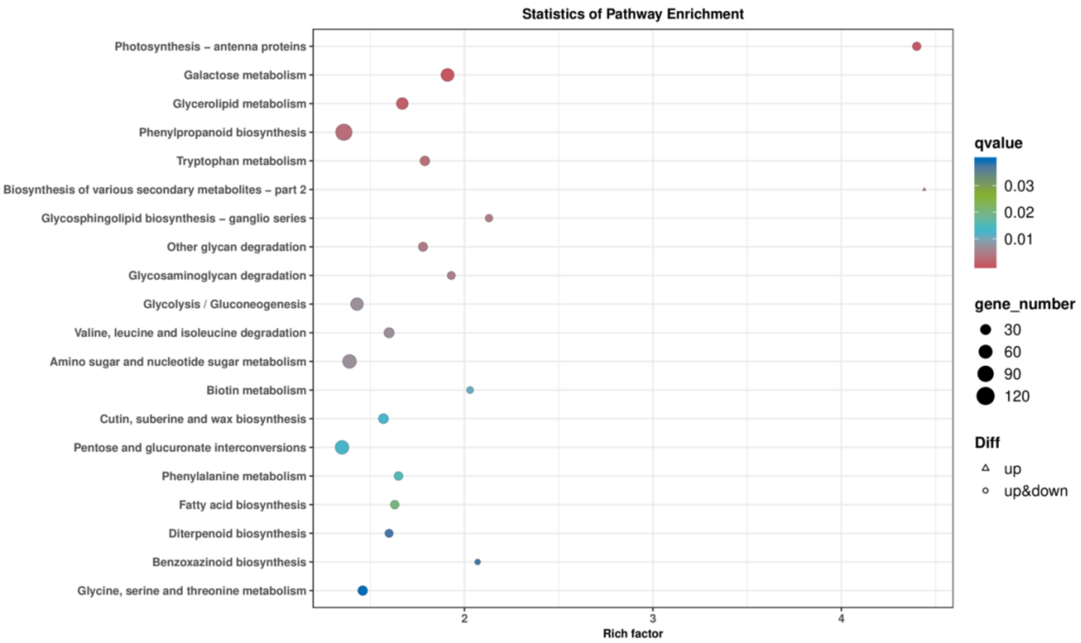

Figure S9. KEGG enrichment analysis of differentially expressed genes 35 day after pollination by between Qiemen and 129-1.

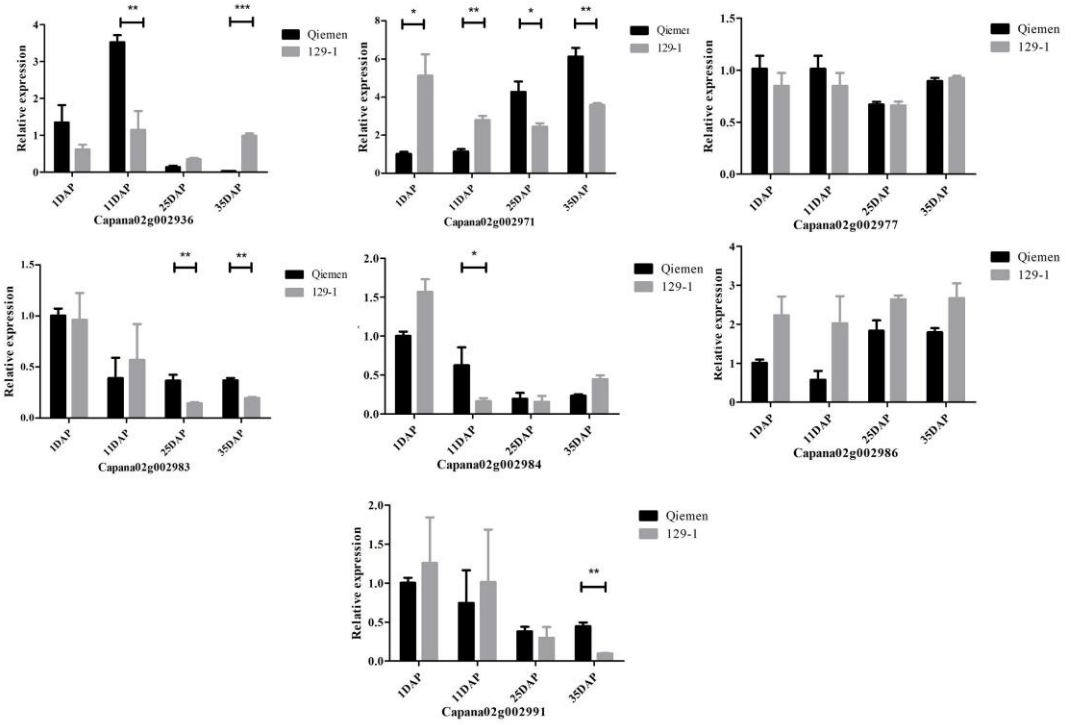

Figure S10. Expression patterns of other candidate genes in *qFW3.1* region.

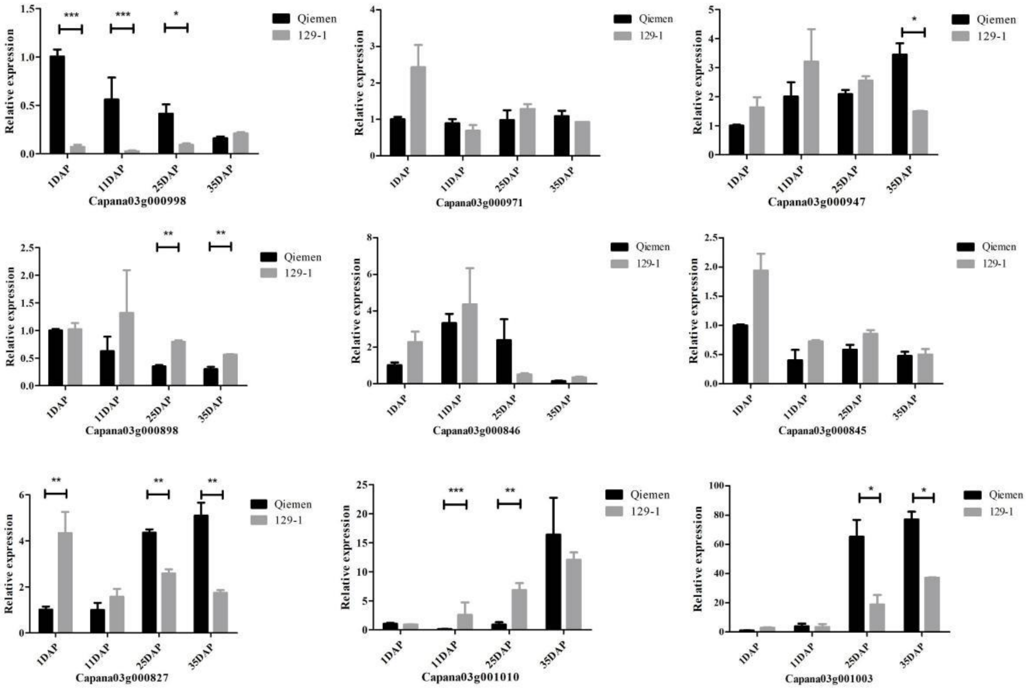

Figure S11. Expression patterns of other candidate genes in *qFW3.1* region.
